# Delivery Care During the SARS-CoV-2 Epidemic in Italy: An Instant Survey

**DOI:** 10.1017/dmp.2020.250

**Published:** 2020-07-14

**Authors:** Elsa Viora, Claudio Crescini, Carlo Maria Stigliano, Fabio Parazzini, Antonio Chiantera

**Affiliations:** Segreteria Nazionale AOGOI, Milan, Italy; Department of Clinical Sciences and Community Health, University of Milan, Italy

**Keywords:** COVID 19, delivery, protection

On February 20, 2020, the first case of severe pneumonia due to severe acute respiratory syndrome coronavirus 2 (SARS-CoV-2) was diagnosed in Italy. Since then, the cases of infection identified in Italy rapidly increased.

Following the epidemic breakthrough, the health authorities developed recommendations and clinical guidance for pregnancy and delivery care for women who tested positive and negative for SARS-CoV-2 during the epidemic period.^1,2^ These guidelines generally provide information on the adopting of protocols for the triage of pregnant and delivering women to identify those with suspected SARS-CoV-2 infection, and the identification of regional designated COVID-19 maternity hospitals (hub hospital) to offer adequate assistance and epidemiological surveillance to symptomatic infected women. However, all hospitals (spoke hospitals) had to be able to organize a protected vaginal or cesarean delivery. This means that appropriate personal protective equipment (PPE) and dedicated labor and delivery or operating rooms had to be available.

To gain a perspective of pregnancy and delivery care in Italy during the COVID-19 epidemic and the procedures for protecting pregnant women and health care providers (HCPs), AOGOI (Associazione Ostetricici e Ginecologi Ospedalieri Italiani, the main Italian gynecological association) has conducted an instant survey.

Data were collected through anonymous online questionnaires. Italian registered gynecologists associated with AOGOI were asked to participate through an announcement published on the website and the weekly newsletter of the AOGOI. The questionnaire was available online between March 20 and April 7, 2020. Data were collected using the SurveyMonkey platform. A total of 332 completed questionnaires were received. Table 1 shows the main results.

**TABLE 1:** Distribution of Answers to the Questionnaire

	N = 332	% (95% CI)
Written protocol for triage/diagnosis/treatment of women with suspected or confirmed SARS-CoV-2 infection (Yes)	247	74.4 (69.3−79.0)
Place of outpatient obstetric visit/ultrasound examination		
Hospital	232	69.9 (64.6−74.8)
Hospital dedicated outpatient services	44	13.3 (9.8−17.4)
Outpatient services	56	16.9 (13.0−21.3)
Adequate availability of PPE		
Surgical mask (Yes)	173	52.1 (46.6−57.6)
All PPE (respirator N95 or FFP2 standard or equivalent, gown, gloves, eye protection, apron)	119	35.8 (30.7−41.3)
Adequate advice on proper use of various PPE (Yes)	250	75.3 (70.3−79.8)
HCP screening for SARS-CoV-2 infection		
None	152	45.8 (40.3−51.3)
Symptoms or contact with COVID-19 patients, or both	160	48.2 (42.7−53.7)
All HCPs	10	3.0 (1.5−5.5)
On request of the HCP	10	3.0 (1.5−5.5)

HCP = health care provider; PPE = personal protective equipment.

A total of 247 responders (74.4, 95% confidence interval (CI): 69.3-79.0) reported that written protocols on the diagnostic and therapeutic procedures for pregnant women with suspected or confirmed SARS-CoV-2 infection were available in their centers. The gynecologists reported that “hub” hospitals were defined in most regions within 3 weeks of the first Italian identified case of COVID-19 (between February 22 and March 26, in most cases, during the period of March 1-16; data not shown in table).

With regard to the availability of PPE, 173 responders (52.1, 95% CI: 46.6-57.6) reported adequate availability of only surgical masks. Respirators (N95) or FFP2 face masks or the equivalent, gown, gloves, eye protection, and apron were reported as adequately available by 119 gynecologists (35.8, 95% CI: 30.7-41.3).

In particular, 78% (259 responders, 95% CI: 73.2-82.4) of gynecologists considered the availability of PPE as inadequate in the delivery and operating rooms (data not shown). A total of 250 gynecologists (75.3%, 95% CI: 70.3-79.8) reported that instructions on the proper use of various PPE were adequate.

Finally, we asked about screening procedures using rRT-PCR tests for SARS-CoV-2 infection among HCPs. Only 10 gynecologists (3.1%) reported that all HCPs were tested. Most of the gynecologists reported that no HCP was tested.

In conclusion, the general results of this instant survey show that adequate procedures were quickly available in Italy for pregnancy and delivery care during the SARS-CoV-2 infection epidemic. Most delivery hospitals have developed specific procedures, and Italian regions have identified hub centers. However, PPE was not adequately available in most of the delivery rooms, and HCPs were not screened for infection.

## References

[1] Ferrazzi EM , Frigerio L , Cetin I , et al. COVID- 19 Obstetrics Task Force, Lombardy, Italy: executive management summary and short report of outcome. Int J Gynaecol Obstet. 2020;epub, doi: 10.1002/ijgo.13162. PMC9087792 32267531

[2] Commissione consultiva tecnico-scientifica sul percorso nascita. Nuovo coronavirus SARS-CoV-2 Indicazioni per le professioniste e i professionisti del percorso nascita della regione Emilia- Romagna. 2020 https://www.sigo.it/wp-content/uploads/2020/03/emilia-romagna-allegato-indicazioni-covid19.pdf. Accessed March 22, 2020.

